# Neuronal Dysfunction and Behavioral Abnormalities Are Evoked by Neural Cells and Aggravated by Inflammatory Microglia in Peroxisomal β-Oxidation Deficiency

**DOI:** 10.3389/fncel.2018.00136

**Published:** 2018-05-23

**Authors:** Lien Beckers, Stijn Stroobants, Rudi D’Hooge, Myriam Baes

**Affiliations:** ^1^Laboratory for Cell Metabolism, Department of Pharmaceutical and Pharmacological Sciences, KU Leuven—University of Leuven, Leuven, Belgium; ^2^Department of Biological Psychology, Faculty of Psychology and Educational Sciences, KU Leuven—University of Leuven, Leuven, Belgium

**Keywords:** microglia, microgliosis, neuron-microglia communication, neurotransmission, peroxisomes, β-oxidation, metabolic disorder, behavior

## Abstract

It is becoming evident that microglia, the resident immune cells of the central nervous system (CNS), are active contributors in neurological disorders. Nevertheless, the impact of microgliosis on neuropathology, behavior and clinical decline in neuropathological conditions remains elusive. A mouse model lacking multifunctional protein-2 (MFP2), a pivotal enzyme in peroxisomal β-oxidation, develops a fatal disorder characterized by motor problems similar to the milder form of human disease. The molecular mechanisms underlying neurological decline in men and mice remain unknown. The hallmark of disease in the mouse model is chronic proliferation of microglia in the brain without provoking neuronal loss or demyelination. In order to define the contribution of *Mfp2^−/−^* neural cells to development of microgliosis and clinical neuropathology, the constitutive *Mfp2*^−/−^ mouse model was compared to a neural selective *Nestin-Mfp2*^−/−^ mouse model. We demonstrate in this study that, in contrast to early-onset and severe microgliosis in constitutive *Mfp2*^−/−^ mice, *Mfp2*^+/+^ microglia in *Nestin-Mfp2*^−/−^ mice only become mildly inflammatory at end stage of disease. *Mfp2*^−/−^ microglia are primed and acquire a chronic and strong inflammatory state in *Mfp2*^−/−^ mice whereas *Mfp2*^+/+^ microglia in *Nestin-Mfp2*^−/−^ mice are not primed and adopt a minimal activation state. The inflammatory microglial phenotype in *Mfp2*^−/−^ mice is correlated with more severe neuronal dysfunction, faster clinical deterioration and reduced life span compared to *Nestin-Mfp2*^−/−^ mice. Taken together, our study shows that deletion of MFP2 impairs behavior and locomotion. Clinical decline and neural pathology is aggravated by an early-onset and excessive microglial response in *Mfp2*^−/−^ mice and strongly indicates a cell-autonomous role of MFP2 in microglia.

## Introduction

Peroxisomal multifunctional protein-2 (MFP2) deficiency is a rare metabolic disorder with important central nervous system (CNS) involvement. Dependent on the type of mutation, patients either present with a neurodevelopmental disorder leading to death within the first year of life (Ferdinandusse et al., 2006) or with a varying milder phenotype. This encompasses a spectrum of clinical presentations ranging in onset from early childhood to adulthood, comprising sensorineural hearing loss, leukodystrophy, intellectual decline, ataxia and sensorimotor neuropathy (Ferdinandusse et al., 2006; Khan et al., 2010; Pierce et al., 2010; Lines et al., 2014). The *Mfp2^−/−^* mouse model rather mimics the milder form of disease and develops a progressive fatal phenotype characterized by motor problems, ataxia, weight loss, and lethargy (Huyghe et al., 2006c; Verheijden et al., 2013). The mechanisms underlying pathology in men and mice are unknown.

MFP2, encoded by the gene *HSD17B4*, is the key enzyme in peroxisomal β-oxidation, a pathway responsible for chain shortening of carboxylates including fatty acids (Huyghe et al., 2006a). In contrast to the mitochondrial β-oxidation pathway, it does not serve energy generation but it is necessary to maintain homeostasis of bioactive lipids. Despite the essential role of MFP2 in the mammalian brain, detailed information on its distribution pattern is lacking. In the human brain, MFP2 immunoreactivity was apparent in both neurons and glial cells at an early fetal stage and increased in late fetal to postnatal stages (Itoh et al., 1999). In murine brain, *in situ* hybridization revealed high expression levels of MFP2 transcripts in hippocampus, thalamus, and cortical subplate but low levels in pons, medulla, and cerebellar regions (Sunkin et al., 2013). However, the role of MFP2 in distinct brain cell types remains unknown.

The hallmark of disease in the *Mfp2^−/−^* mouse model is development of extensive microgliosis in gray matter regions of the CNS in the absence of overt neuronal loss and demyelination (Verheijden et al., 2013, 2015; Beckers et al., 2017). We recently characterized the unique phenotype of microglia in *Mfp2*^−/−^ mice and found that they strongly proliferate, adopt a chronic and primed activation state and lose their typical homeostatic signature (Verheijden et al., 2015). They obtain a mixed pro- and anti-inflammatory phenotype, but some mediators considered to be detrimental for neurons such as iNOS are not increased, in accordance with the absence of phagocytic activities (Verheijden et al., 2013, 2015). There is no infiltration of peripheral monocytes, no systemic inflammation and no contribution of peripheral pathology to the clinical deterioration in *Mfp2*^−/−^ mice (Jia et al., 2003; Huyghe et al., 2006b,c; Verheijden et al., 2013, 2015). We previously generated a mouse model in which the HSD17B4 gene was conditionally inactivated in neural progenitor cells (*NestinCre^+^Mfp2^loxP/loxP^*, further denoted as *Nestin-Mfp2*^−/−^ mice) resulting in the complete depletion of the enzyme in neurons, astrocytes and oligodendrocytes (Verheijden et al., 2013, 2014). Whereas constitutive *Mfp2*^−/−^ mice (further denoted as *Mfp2*^−/−^) develop chronic and extensive microgliosis and die within 4–6 months, *Nestin-Mfp2*^−/−^ mice only develop minor microgliosis and survive up to 12 months. In contrast to the severe lethargic pathology in *Mfp2*^−/−^ mice, *Nestin-Mfp2*^−/−^ mice develop a milder clinical decline dominated by ataxia at a preterminal stage of disease (Verheijden et al., 2013, 2014).

Microglia, the resident immune cells of the CNS, critically contribute to brain homeostasis both during development and adulthood (Wake et al., 2009; Tremblay et al., 2010; Paolicelli et al., 2011; Schafer et al., 2012; Salter and Beggs, 2014). Proper bidirectional neuron-microglia signaling is essential to fine-tune fundamental processes in the healthy CNS (Prinz and Priller, 2014; Yirmiya et al., 2015; Rohan Walker and Yirmiya, 2016). Microglia create a neuroprotective environment by releasing neurotrophic factors and anti-inflammatory cytokines in physiological conditions (Elkabes et al., 1996; Batchelor et al., 2002; Coull et al., 2005; Vukovic et al., 2012; Béchade et al., 2013). Vice versa, neuronal suppressive signals including CX3CL (fractalkine) and CD200 restrain microglial activation in the healthy brain (Polazzi and Monti, 2010; Saijo and Glass, 2011; Li et al., 2012; Eyo and Wu, 2013). Aberrant neuron-microglia interactions in neuropathological conditions such as loss of neuronal restraint on microglia due to neuronal dysfunction or degeneration can trigger microglial activation (Ransohoff and Cardona, 2010; Saijo and Glass, 2011; Wolf et al., 2013; Biber et al., 2014; Cardona et al., 2015). Activation of microglia in CNS disease is often described as a double-edged sword as they mediate both neuroprotective and neurotoxic effects (Block et al., 2007; Biber et al., 2014; Prinz and Priller, 2014; Crotti and Ransohoff, 2016). Dependent on timing and type of disease, microglial activation can be cause, contributor, bystander, protector or consequence of neuronal dysfunction.

We hypothesized that the severe phenotype and shorter life span of *Mfp2^−/−^* compared to *Nestin-Mfp2*^−/−^ mice is related to the robust microglial reactivity in *Mfp2*^−/−^ mice. We therefore characterized the microglial features of both mouse models and determined whether constitutive vs. neural-selective MFP2 deficiency differentially affects neurological function in order to illuminate the cellular mechanisms of disease. Hereto, brainstem auditory evoked potentials (BAEPs), explorative locomotion, and grip strength were assessed in early, mid and late stages of disease in both models.

## Materials and Methods

### Mouse Breeding

The generation and characterization of* Mfp2^−/−^* and *Nestin-Mfp2*^−/−^ mice has been described (Baes et al., 2000; Verheijden et al., 2013). *Mfp2^loxP/loxP^* mice in which exon 8 of the *HSD17B4* gene is flanked by LoxP sequences were bred with *Nestin-Cre* mice (Tronche et al., 1999) that cause recombination in neural progenitor cells that give rise to neurons, oligodendrocytes and astrocytes. *Mfp2*^−/−^ and *NestinCre^+^Mfp2^loxP/loxP^* (denoted as *Nestin-Mfp2*^−/−^ mice) were bred on a Swiss/Webster background. As we did not detect differences between wild type and heterozygous mice in our previous investigations, both were used as controls for *Mfp2*^−/−^ and *Nestin-Mfp2*^−/−^ mice. Genotyping was performed on tail DNA. All mice were bred in the animal housing facility of the KU Leuven, had *ad libitum* access to water and standard rodent food, and were kept on a 12-h light and dark cycle. This study was carried out in accordance with the recommendations of “Guidelines for Care and Use of Experimental Animals” and fully approved by the Research Ethical committee of the KU Leuven (#190/2012, #181/2015).

### Murine Behavioral Studies

#### Auditory Brainstem Response Test

BAEPs were recorded on a Myos 4 plus digital EMG/EP recorder (Schwarzer, Munich, Germany). Mice were anesthetized with Nembutal (6 mg/ml i.p. at 1% body weight). A needle electrode was placed subcutaneously behind each ear and referenced to a common electrode near the base of the tail; a fourth electrode, also placed near the base of the tail, was used as a ground (Willott, 2006). Robust five-peak tracings were obtained by averaging 2000 responses evoked by 85–86 dB clicks (measured with a Brüel & Kjær (Norcross, GA, USA) sound intensity meter) emitted by a speaker placed 1 cm in front of the animal’s head. For each of the tracings, latencies of the first five peaks (numbered I through V) were measured as well as interpeak latencies I–III, III–V, and I–V. In addition, mean amplitudes of all peaks were measured.

#### Exploration by Open Field (OF) Assessment

OF exploration was tested in a brightly illuminated 50 cm × 50 cm square arena subsequent to 30 min of dark adaptation (Seibenhener and Wooten, 2015). Movements in the arena were video-tracked for 10 min. Total path length, distance to the center and number of corner entries were assessed.

#### Grip Strength Measurement

The grip strength test measures either the forepaw strength or a combined forepaw and hind paw strength. Mice were allowed to grasp the triangular grid on the grip strength meter with their forepaws only and were then gently pulled from the base of the tail until they released the grip. The grip strength meter measures the maximum force applied to the meter (Smith et al., 1995). This was repeated five times per mouse. Grip strength of combined forepaw and hind paw strength was measured by repeating the protocol but placing the mouse with four paws on the rectangular grid before pulling it gently from the base of the tail.

### Immunohistochemistry (IHC)

Mice were anesthetized with a mix of Dormitor (1 mg/kg) and Nimatek (75 mg/kg). Tissue processing and IHC staining were performed as described (Huyghe et al., 2006c; Hulshagen et al., 2008; Bottelbergs et al., 2012). Briefly, mice were perfused transcardially with PBS (pH 7.4) followed by 4% paraformaldehyde (PFA). Brains were isolated, post-fixed with 4% PFA overnight, and kept in 70% ethanol prior to paraffin embedding. Routinely, paraffin sections (7 μm) were used for immunofluorescent stainings. The following primary antibodies were used: polyclonal rabbit anti-Iba1 (1:500; Wako D19-19741), rat anti-F4/80 (1:500; Serotec, Oxford, UK). Cryo sections were used for staining with polyclonal rabbit anti-P2ry12 (1:500; provided by Prof. O. Butovsky, Boston, MA, USA). After incubation with primary antibodies overnight at room temperature (at 4°C for anti-P2ry12 staining) HRP-labeled secondary antibodies (1:200) were applied for 1 h, followed by fluorescent labeling with a cyanine 2 (FITC) TSA kit (Perkin Elmer Life Sciences, Boston, MA, USA). P2ry12 antibodies were detected with goat anti-rabbit IgG conjugated to Alexa647 (1:300; Life Technologies A21244). When double immunolabeling was performed, sets of primary and secondary antibodies were applied sequentially. As second fluorescent labels, cyanine 3 TSA kits (Perkin-Elmer) were used. Images were acquired with a motorized inverted IX-81 microscope connected to a CCD-FV2T digital camera (Olympus, Aartselaar, Belgium) and processed with LSM Image browser software (Zeiss, Germany).

### Quantification of IHC

#### Cell Number Quantification

Microglial cell numbers were quantified on paraffin sections (7 μm) after immunofluorescent staining with anti-Iba1 antibodies. Iba1^+^ cells in the CNS were shown to be resident microglia as they express microglia-specific markers such as P2ry12 and as we showed previously that there is no influx of peripheral cells in the CNS of *Mfp2^−/−^*, *Nestin-Mfp2*^−/−^ and control mice (Verheijden et al., 2015). Cells were counted around the sagittal midline and coronal plane at the height of brainstem, cortex and colliculus area. Within one plane (20× magnification), only Iba1-positive cells that: (1) fully co-localized with DAPI-positive nuclei; and (2) had a clear cell soma; and (3) had at least two clear protrusions were counted in different regions of the brain. Microglial number per frame was corrected for surface area. Three to five different pictures per brain region per mouse were taken. The number of microglia was counted and the average of all pictures per brain region was used to quantify the number of microglial cells per brain region per mouse model. Both *Mfp2*^−/−^, *Nestin*-*Mfp2*^−/−^ and their respective control mice at the ages of 3, 6, 12, 17 and 34 weeks were used (*n* = 4–6/group).

#### Fluorescence Intensity Quantification

P2ry12 immunofluorescence intensity was measured in different brain regions such as brainstem, visual cortex and inferior colliculus within one plane of 4× magnification. Using the ImageJ software (Fiji), color channels were first split, background signal was subtracted by selecting an area of the section that expressed noise fluorescence, the area of interest was selected, and mean gray value was subsequently measured at the same area size per brain region. Three to five pictures per brain region per mouse were quantified and the average of these pictures was used to calculate the P2ry12 immunofluorescence intensity per brain region per mouse model (*n* = 3–4/group).

### Flow Cytometry

Microglia cells were isolated by mechanical dissociation and Percoll gradient centrifugation as previously described (Verheijden et al., 2015). For immunological profiling, 1 × 10^5^ cells were incubated for 20 min at 4°C using combinations of CD11b-Pe Cy7 (clone M1/70, Biolegend) with either CD11c-APC (clone HL3, BD Pharmingen), F4/80-APC (clone BM8, eBioscience), CD204-Alexa 647 (clone MR5D3, AbD Serotec), CD206-Alexa 647 (clone 2F8, AbD Serotec) antibodies all at dilutions of 1:200 in a total volume of 200 μl. Flow cytometry was performed on a FACS Verse (BD Biosciences), and data was analyzed with BD FACsuite software (BD Biosciences). Injection and subsequent analysis of Bromodeoxyuridine (BrdU; FITC BrdU Flow Kit 559619, BD Biosciences) in mice by flow cytometric analysis has been described (Verheijden et al., 2015).

### Real-Time Quantitative PCR (RT-qPCR)

Regarding analysis of primed microglia, injection of Lipopolysaccharides (LPS) and vehicle in mice and subsequent brain sampling has been described (Verheijden et al., 2015). Total RNA was isolated from brain tissue using Trizol reagent (Invitrogen, Carlsbad, CA, USA). Subsequently, cDNA was generated from 1 μg RNA using the QuantiTect Reverse Transcription Kit (QIAGEN, Venlo, Netherlands). Real time PCR was performed as previously described (Bottelbergs et al., 2012) using an ABI PRISM 7500 Real Time PCR instrument (Applied Biosystems, Lennik, Belgium). Predesigned and validated primers and probes were ordered from Applied Biosystems as premade Taqman Gene Expression assays (Il1b, Mm011336189_m1; Cx3cr1, Mm0262011_s1; Tgfbr1, Mm00436964_m1; cd200, Mm.PT.58.33215550; Cx3cl1, Mm.PT.58.8767901). Assays were performed in duplicate or triplicate in 10 μL TaqMan Fast Universal PCR Master Mix (Applied Biosystems). Relative expression levels of the target genes were calculated taking into account the amplification efficiency as described (Giulietti et al., 2001). The relative expression levels of the target genes were calculated as a ratio to housekeeping genes such as β-actin.

### Statistical Analysis

All data except some behavioral tests (see below) were analyzed with GraphPad Prism software (version 5.0 and 6.0, San Diego, CA, USA). Statistical analyses were carried out using unpaired and paired, two-sided Student’s *t*-test, one-way ANOVA, two-way ANOVA or two-way repeated measures (RM) ANOVA followed by the Bonferroni *post hoc* test. Data are shown as mean ± Standard Error of the Mean (SEM) and statistical significance was set at *p* < 0.05. SPSS Statistics software was used for three-way ANOVA and three-way RM ANOVA.

#### BAEP Test

Three-way RM ANOVA with genotype, age, and peak as sources of variation and two-way RM ANOVA per age with genotype and peak as sources of variation were used to evaluate disease progression of *Mfp2^−/−^* and *Nestin*-*Mfp2*^−/−^ mice. Subsequently, performance of the knockout mice (expressed as relative to their respective control values) was directly compared using three-way ANOVA for analyses of peak latencies, with age and model as independent variables and peak as dependent variable. Two-way ANOVA was used for evaluation of interpeak latencies, with age and model as independent variables.

#### OF Test

Two-way ANOVA with genotype and age as sources of variation was used to evaluate disease progression of *Mfp2^−/−^* and *Nestin*-*Mfp2*^−/−^ mice. Subsequently, performance of *Mfp2*^−/−^ and *Nestin*-*Mfp2*^−/−^ mice (expressed relative to their respective control values) was directly compared using two-way ANOVA with age and model as independent variables.

The Holm-Sidak and Bonferroni method was used for multiple comparisons. Independent samples *t*-test was used to compare performance at pathological end stages.

## Results

### Early-Onset and Rapid Increase in Microgliosis in *Mfp2^−/−^* Mice vs. Late-Onset and Minor Microgliosis in *Nestin-Mfp2^−/−^* Mice

**Figure 1:**
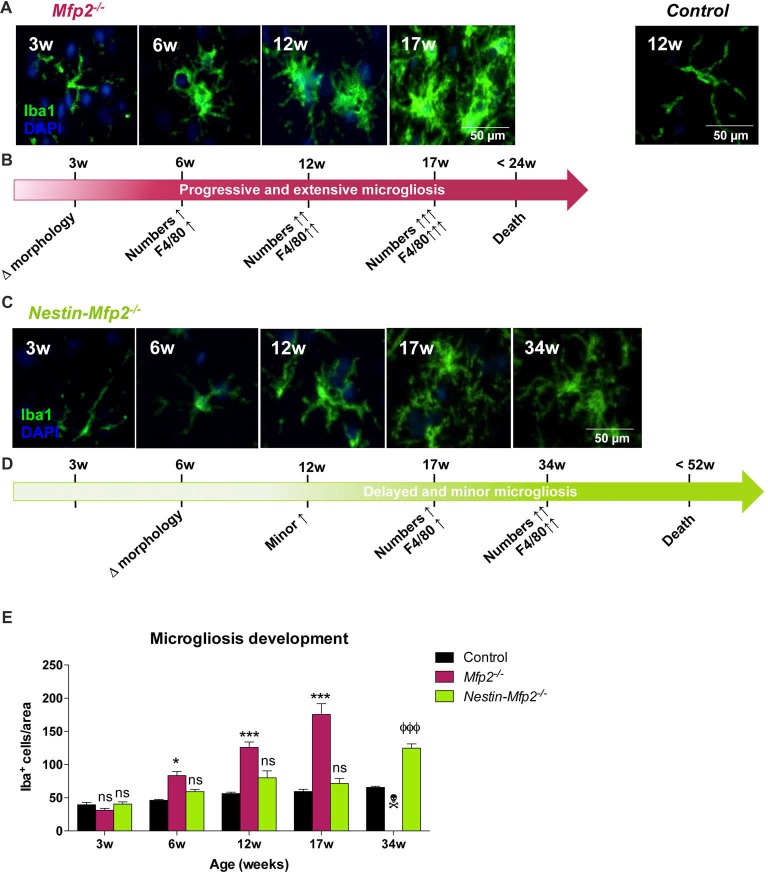
Early onset and extensive microgliosis in Multifunctional protein-2 (*MFP2*^−/−^) vs. limited and delayed microgliosis in *Nestin-Mfp2*^−/−^ mice. **(A,B)** Age-dependent progression of microgliosis in brainstem of *Mfp2*^−/−^ mice. **(A)** Morphology of *Mfp2*^−/−^ microglia (Iba1^+^) at different time points. **(B)** Timeline showing progression of microglial transformation (see **A**), proliferation (see **E**) and F4/80 activation (based on immunohistochemistry (IHC), see Figure 2A) in *Mfp2*^−/−^ brain. **(C,D)** Age-dependent progression of microgliosis in brainstem of *Nestin-Mfp2*^−/−^ mice. **(C)** Morphology of microglia in *Nestin-Mfp2*^−/−^ brain at different time points. **(D)** Timeline showing progression of microglial transformation (see **C**), proliferation (see **E**) and F4/80 activation (based on IHC, see Figure 2A) in *Nestin-Mfp2*^−/−^ brain. **(E)** Quantification of Iba1^+^ cells (microglia) in *Mfp2*^−/−^, *Nestin-Mfp2*^−/−^ and control brainstem. w, weeks. *Mfp2^−/−^ vs. control: *p < 0.05, ***p < 0.001. ^Ф^*Nestin-Mfp2*^−/−^ vs. control: ^ФФФ^*p* < 0.001, ns, not significant. Error bars indicate standard error of the mean (SEM). *n* = 6–9/group. 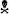
*Mfp2*^−/−^ mice all died at this time point.

In order to define the onset and extent of microglial reactivity in constitutive *Mfp2^−/−^* and conditional *Nestin*-*Mfp2*^−/−^ mice, we characterized microglial cell numbers and morphology at different time points (at the age of 3, 6, 12, 17 and 34 weeks; Figures 1A–D). *Mfp2*^−/−^ mice could only be observed till 17 weeks as they die from 16 weeks of age onwards whereas *Nestin*-*Mfp2*^−/−^ mice survive up to 48 weeks.

At 3 weeks of age, we found that most Iba1^+^ microglial cells displayed a healthy “resting” morphology characterized by a small cell soma and long and fine protrusions in both mouse models (Figure 1A). A few microglial cells with larger soma and thicker protrusions, defined as “activated” or inflammatory microglia, were locally observed in *Mfp2^−/−^* mice. At this time point, no significant differences in microglial cell numbers were observed compared to control mice (Figure 1E, shown for brainstem). At 6 weeks of age, microglia in *Mfp2*^−/−^ mice displayed a morphology with larger cell soma and more and thicker protrusions in some brain regions such as brainstem and inferior colliculus. *Nestin*-*Mfp2*^−/−^ microglia remained unaffected (Figure 1C). At this age, numbers of microglia were significantly increased in different brain regions of *Mfp2*^−/−^ but not of *Nestin*-*Mfp2*^−/−^ mice (Figure 1E). Microgliosis expanded at 12 weeks and culminated at 17 weeks in all brain regions in *Mfp2*^−/−^ mice whereas it remained restricted in *Nestin*-*Mfp2*^−/−^ mice at these ages. The numbers of microglia clearly increased at 34 weeks of age in *Nestin*-*Mfp2*^−/−^ mice, a time point when all *Mfp2*^−/−^ mice had died (Figure 1E). Microglia in *Nestin*-*Mfp2*^−/−^ mice underwent a morphological transformation at end stage of disease but never acquired the swollen cell soma and thick protrusions as observed in *Mfp2*^−/−^ microglia (Figures 1A,C). We previously demonstrated that increased microglia numbers in *Mfp2*^−/−^ mice derive from strong proliferation of resident microglia and not from infiltration of peripheral immune cells (Verheijden et al., 2015). By means of an *in vivo* BrdU incorporation experiment and subsequent flow cytometric analysis, we verified that increased numbers of Iba1^+^ cells at later ages in *Nestin*-*Mfp2*^−/−^ mice were also proliferating resident microglia (Data not shown). Taken together, proliferation and morphological changes of microglial cells are delayed in time and limited in *Nestin*-*Mfp2*^−/−^ mice compared to *Mfp2*^−/−^ mice. Microgliosis in the *Nestin*-*Mfp2*^−/−^ brain (34 weeks) is never as extensive as in preterminal (17 weeks) *Mfp2*^−/−^ brain.

### Microglia in *Nestin-Mfp2^−/−^* Brain Acquire a Mild Inflammatory Activation State Compared to *Mfp2^−/−^* Microglia

During neuropathology, microglia lose their homeostatic molecular signature and adopt an inflammatory activated phenotype (Butovsky et al., 2014, 2015; Perry and Holmes, 2014; Krasemann et al., 2017). Transcriptomics on microglia from *Mfp2^−/−^* mice revealed a coordinated loss of homeostatic markers during disease (Verheijden et al., 2015). It remains unknown whether adaptation of the microglial signature in MFP2 deficiency is a reaction to ongoing neural pathology caused by absence of MFP2 from neural cells. Therefore, we compared typical homeostatic and inflammatory microglial markers in *Mfp2*^−/−^ brain to neural-specific *Nestin-Mfp2*^−/−^ brain.

**Figure 2:**
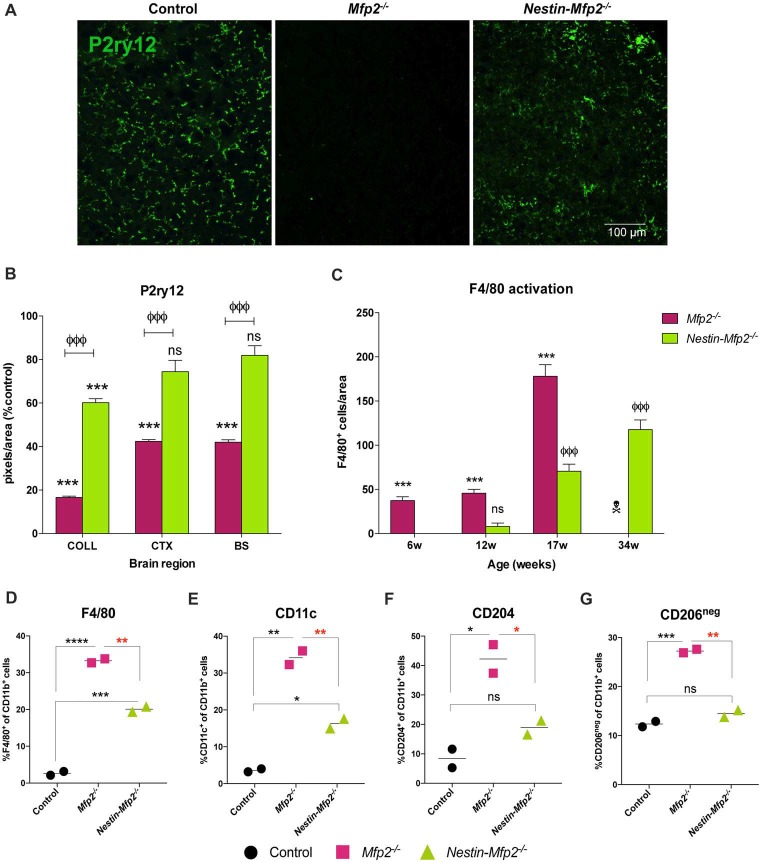
Microglia in *Nestin-Mfp2^−/−^* brain acquire a milder inflammatory state than *Mfp2*^−/−^ microglia. **(A)** Representative immunohistochemical staining for P2ry12 in inferior colliculus of 17-weeks-old *Mfp2*^−/−^ mice and age-matched *Nestin-Mfp2*^−/−^ mice relative to control mice. **(B)** Quantification of P2ry12 in 17-weeks-old *Mfp2*^−/−^ mice and *Nestin-Mfp2*^−/−^ mice relative to age-matched control mice in three brain regions. *Mfp2^−/−^ or *Nestin-Mfp2*^−/−^ vs. control: ***p < 0.001, ns, not significant. ^Ф^*Nestin-Mfp*^2−/−^ vs. *Mfp*^2−/−^: ^ФФФ^*p* < 0.001. *n* = 3–6/group. COLL, Inferior Colliculus, CTX, Visual Cortex, BS, Brainstem. **(C)** F4/80^+^ cells were counted in brainstem of *Mfp2*^−/−^, Nestin-Mfp2^−/−^ and control mice. Graphs from control mice are not shown as they approach zero. w, weeks. *Mfp2^−/−^ vs. control: ***p < 0.001. ^Ф^*Nestin-Mfp2*^−/−^ vs. control: ^ФФФ^*p* < 0.001, ns, not significant. 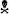
*Mfp2*^−/−^ mice all died at this time point. *n* = 6–9/group. **(D–G)** Flow cytometric analysis of CD11b^+^ microglia isolated from 17-week-old *Mfp2*^−/−^ mice and 34-week-old *Nestin-Mfp2*^−/−^ mice using antibodies to pro-inflammatory markers F4/80 **(D)** and CD11c **(E)**, and anti-inflammatory markers CD204 **(F)** and CD206 **(G)**. Representative figures of multiple independent experiments are shown (Total: *n* = 4/group). *p < 0.05, **p < 0.01, ***p < 0.001, ****p < 0.001, ns, not significant. Error bars indicate SEM.

P2ry12, which has become the hallmark of the homeostatic microglia signature, was strongly suppressed in *Mfp2^−/−^* microglia in all brain areas, confirming previous observations (Figures 2A,B; Butovsky et al., 2014; Verheijden et al., 2015). In contrast, P2ry12 was not downregulated in *Nestin-Mfp2*^−/−^ mice except in the inferior colliculus (Figures 2A,B). In this region, suppression of P2ry12 in microglia of *Nestin-Mfp2*^−/−^ mice was much less pronounced as compared to loss of P2ry12 in *Mfp2*^−/−^ microglia (Figures 2A,B).

Time course of protein expression of the well-known activation marker F4/80 or EMR1 revealed that F4/80^+^ cells in *Mfp2^−/−^* mice were significantly increased at 6 weeks of age and further expanded with age. In contrast, F4/80^+^ cells were significantly elevated in *Nestin*-*Mfp2*^−/−^ mice from 17 weeks of age (Figure 2C). F4/80^+^ microglial cells are virtually absent in control brain at all ages, so bars from control brain are not shown in the graph (Figure 2C). Of note, F4/80^+^ cells in brain of *Mfp2*^−/−^ mice are assigned as inflammatory microglia as there is no influx of peripheral cells in the CNS (Verheijden et al., 2015). We previously demonstrated that *Mfp2*^−/−^ microglia are inflammatory activated but not skewed into a polarized pro- or anti-inflammatory state (Verheijden et al., 2015). We now isolated microglia of *Nestin-Mfp2*^−/−^ and age-matched control brain at 34 weeks of age to reveal their activation state by flow cytometry. For direct comparison, *Mfp2*^−/−^ microglia were isolated and analyzed for the same markers in the same experiment (at 17 weeks of age as *Mfp2*^−/−^ mice die prematurely). Pro-inflammatory surface markers CD83 and CD86, which are induced in *Mfp2*^−/−^ microglia, were not altered in microglia of *Nestin-Mfp2*^−/−^ mice compared to microglia of control mice (not shown, Verheijden et al., 2015). Other pro-inflammatory markers such as F4/80 and CD11c were induced in *Nestin-Mfp2*^−/−^ microglia compared to control microglia but did not attain the levels as in *Mfp2*^−/−^ microglia (Figures 2D,E). In contrast to significant induction in *Mfp2*^−/−^ microglia, the anti-inflammatory markers CD204 and CD206 were not significantly changed in *Nestin-Mfp2*^−/−^ microglia (Figures 2F,G).

We conclude that inflammatory activation of microglial cells in *Nestin-Mfp2^−/−^* mice is mild and delayed in time compared to early and strong activation of *Mfp2*^−/−^ microglia. Microglia in *Nestin-Mfp2*^−/−^ mice never acquire the highly inflammatory state of *Mfp2*^−/−^ microglia (Verheijden et al., 2015). This shows that the highly inflammatory state of microglia in *Mfp2*^−/−^ mice cannot be attributed to neural pathology elicited by loss of MFP2 in neural cells.

### Microglia Are Not Primed in *Nestin-Mfp2^−/−^* Mice in Contrast to *Mfp2^−/−^* Microglia

**Figure 3:**
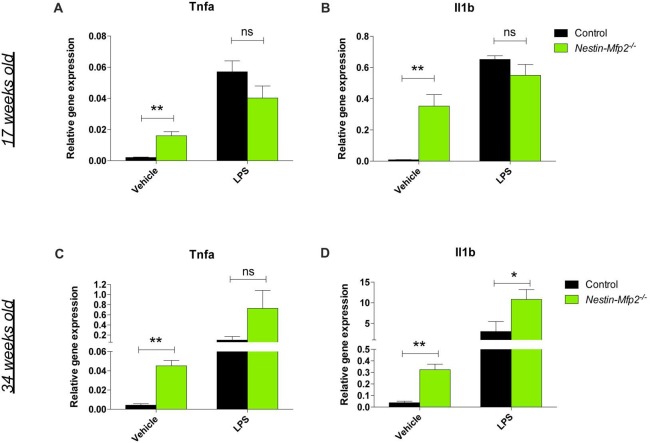
Microglia in *Nestin-Mfp2^−/−^* brain are not primed. *Nestin-Mfp2*^−/−^ and control mice were challenged with i.p. lipopolysaccharides (LPS) or vehicle, and brainstem tissue was analyzed after 4 h. **(A,B)** Transcript levels of *Tnfa* **(A)** and *Il1b* **(B)** in 17-week-old *Nestin-Mfp2*^−/−^ and age-matched control mice after LPS treatment. **(C,D)** Transcripts levels of *Tnfa* **(C)** and *Il1b* levels **(D)** in 34-weeks-old *Nestin-Mfp2*^−/−^ brain compared to control brain after LPS treatment. *Nestin-Mfp2^−/−^ vs. control: *p < 0.05, **p < 0.01, ns, not significant. Error bars indicate SEM. *n* = 4–6/group.

A recently recognized phenomenon in neurodegeneration is priming of microglia. Microglia are initially triggered by a first stimulus in their CNS environment such as a pathological insult or aging. Upon a secondary inflammatory stimulus, primed microglia release excessive quantities of pro-inflammatory cytokines (Perry and Holmes, 2014). We previously showed that *Mfp2^−/−^* microglia are primed as they exhibit an excessive induction of *Tnfa* and *Il1b* transcript and protein levels after i.p. injection of LPS (Verheijden et al., 2015). This treatment was also administered to *Nestin-Mfp2*^−/−^ mice in order to reveal whether microglia in a MFP2 deficient neural environment are also primed. In contrast to the excessive inflammatory response of *Mfp2*^−/−^ microglia to LPS, microglia in 17-weeks-old *Nestin-Mfp2*^−/−^mice respond similarly as microglia in age-matched control mice based on *Tnfa* and *Il1b* transcript levels (Figures 3A,B). Nevertheless, microglia in *Nestin-Mfp2*^−/−^ mice are inflammatory activated at this age as expression levels of *Tnfa* and *Il1b* are increased in mutant mice as compared to control mice after vehicle treatment, in agreement with the flow cytometry data (Figures 3A,B). Thus, microglia in 17-weeks-old *Nestin-Mfp2*^−/−^ mice are not primed. In order to exclude that priming of microglia in these mice occurs at more advanced stages of pathology, we repeated this experiment at the age of 34 weeks. After LPS treatment, transcript levels of *Tnfa* (7-fold, not significant) and *Il1b* (3.5-fold, *p* < 0.05) were higher in *Nestin-Mfp2*^−/−^ compared to LPS-treated control mice (Figures 3C,D). However, there was no excessive inflammatory response as we previously observed in *Mfp2*^−/−^ microglia i.e., a 40-fold increase (*p* < 0.001) in *Tnfa* and a 10-fold increase (*p* < 0.001) in *Il1b* transcripts vs. LPS-treated controls (Verheijden et al., 2015). Thus, despite clear microgliosis in *Nestin-Mfp2*^−/−^ brain at an advanced stage of pathology, the inflammatory microglia are not primed. These results demonstrate that neural pathology by itself does not induce priming of microglia in *Mfp2*^−/−^ mice.

### Loss of Suppressive Neuronal Signals in *Mfp2^−/−^* But Not in *Nestin-Mfp2^−/−^* Brain

**Figure 4:**
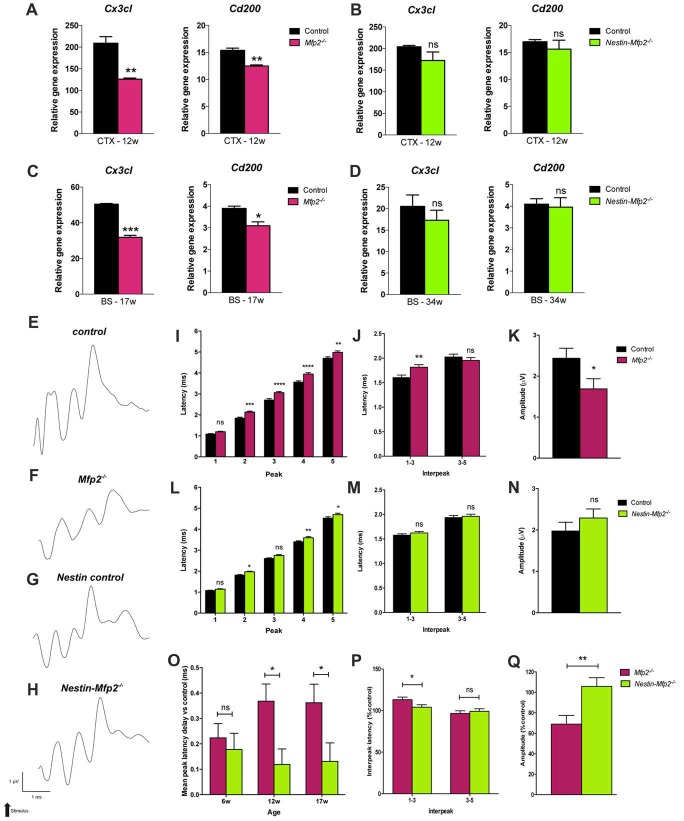
Neuronal functioning is severely impaired in *Mfp2^−/−^* vs. *Nestin-Mfp2*^−/−^ mice.** (A,B)** Transcript levels of neuronal *Cx3cl* and *Cd200* markers in cortex (CTX) of 12-week-old *Mfp2*^−/−^ **(A)** and *Nestin-Mfp2*^−/−^ mice **(B)**. **(C,D)** Transcript levels of neuronal *Cx3cl* and *Cd200* markers in brainstem (BS) of 17-weeks-old *Mfp2*^−/−^ mice **(C)** and 34-week-old *Nestin-Mfp2*^−/−^ mice **(D)**. Transcript levels were measured by qRT-PCR. *n* = 3–6/group.** (E–H)** Wave patterns of the auditory signal from different mouse models are shown. Arrow indicates appearance of acoustic stimulus. Scale bars are shown beneath the bottom tracing. **(I–N)** Brainstem responses on auditory stimulus in *Mfp2*^−/−^ and *Nestin-Mfp2*^−/−^ vs. control mice at 12 weeks of age. **(I,L)** Mean peak latencies show peaks assigned to different brain nuclei (peak 2–5). **(J,M)** Interpeak latencies between peak one to three and peak three to five. **(K,N,Q)** Mean peak amplitudes. **(O–Q)** Direct comparison between *Mfp2*^−/−^ and *Nestin-Mfp2*^−/−^ mice relative to respective control mice at 12 weeks of age. **(O)** Mean peak latency delays vs. control mice (i.e., 0.0 ms) at different stages of disease, **(P)** 1–3 and 3–5 interpeak latencies and **(Q)** mean peak amplitudes in *Mfp2*^−/−^ and *Nestin-Mfp2*^−/−^ mice (Controls are 100%). Significance level: *p < 0.05, **p < 0.01, ***p < 0.001, ****p < 0.0001, ns, not significant. Error bars indicate SEM. *n* = 8–15 mice/group.

A healthy brain is maintained by a bidirectional communication between neuronal cells and microglia whereby neurons chronically restrain microglia and vice versa microglia create a neuroprotective environment (Biber et al., 2014). As proliferative *Mfp2^−/−^* microglia are mainly found in gray matter regions of *Mfp2*^−/−^ brain (Huyghe et al., 2006c; Verheijden et al., 2013), we hypothesized that neuron-microglia signaling is affected. Microglial CX3CR1 is the only receptor for fractalkine (CX3CL1), a neuronal ligand that acts as a critical inhibitory signal to retain microglia in a quiescent state (Wolf et al., 2013). Transcript levels of *Cx3cl1* were not altered at 6 weeks of age (not shown) but were nearly twofold reduced in 9- and 12-weeks-old *Mfp2*^−/−^ brain relative to control brain (Figure 4A). As regards the other inhibitory ligand-receptor pair CD200-CD200R, we found that expression of neuronal ligand *Cd200* was significantly reduced in *Mfp2*^−/−^ brain at the age of 12 weeks but not at younger ages (Figure 4A). Neuron-microglia communication was not impaired in 12-weeks-old *Nestin-Mfp2*^−/−^ brain as expression levels of neuronal markers *Cx3cl1* and *Cd200* were unchanged (Figure 4B).

Neuron-microglia communication was further investigated at final stages of pathology in brainstem, a region that is most severely affected by microgliosis, of *Mfp2^−/−^* (17-weeks-old) and *Nestin-Mfp2*^−/−^ (34-weeks-old) mice. Expression of the neuronal markers *Cx3cl1* and *Cd200* that restrain microglia activation were significantly downregulated in *Mfp2*^−/−^ brain but remained unchanged in *Nestin*-*Mfp2*^−/−^ brain although microgliosis is clearly present at this age (Figures 4C,D). Taken together, impaired signaling of specific neuronal restraint markers (*Cx3cl* and *Cd200*) in *Mfp2*^−/−^ brain but not in *Nestin*-*Mfp2*^−/−^ brain reveals that loss of neuronal restraint signals cannot be attributed entirely to neural dysfunction and indicates a pathological role of *Mfp2*^−/−^ microglia in disturbing neuron-microglia interactions.

### Severe Decline in Brainstem Responses in *Mfp2^−/−^* Mice But Not in *Nestin-Mfp2^−/−^* Mice

The consequences of global or cell type-selective deletion of MFP2 in the brain on the cellular level such as neuronal functionality remain unknown. BAEPs were recorded to analyze neuronal functionality and conductivity by measurement of the auditory signal which is transmitted from the cochlea via specific brainstem nuclei to the auditory cortex. The auditory brainstem responses are depicted as a wave with five peaks corresponding to distinct locations in the auditory pathway, more specifically in the brainstem, an area that is severely affected by microgliosis in our disease model. Peaks represent the response and transmission of the signal by the auditory nerve (peak 1), the response in the cochlear nucleus (peak 2), superior olivary complex (peak 3) and inferior colliculus (peak 4) in brainstem. Peak 5 cannot be precisely assigned as it corresponds to several areas including medial geniculate nucleus in thalamus and the auditory cortex (Melcher and Kiang, 1996; Singer et al., 2014).

BAEP recorded tracings show severe disturbances in the wave pattern of *Mfp2^−/−^* mice whereas the wave pattern of *Nestin-Mfp2*^−/−^ mice was minimally altered as compared to normal evoked brainstem responses in control mice of both genotypes (Figures 4E–H). Peak latencies, reflecting the efficiency of signal transmission and response to an auditory stimulus, were analyzed at the age of 6, 12 (shown in Figures 4I–N), 17 and 34 weeks.

Delayed responses were already evident at the age of 6 weeks in both models but were less pronounced in *Nestin*-*Mfp2*^−/−^ mice. In *Mfp2*^−/−^ brain, the delay observed at the age of 6 weeks aggravated till the age of 12 weeks for peak 1 and 2, and further progressed till 17 weeks of age for peak 1, 2 and 3 (Supplementary Figure S1A), whereas no significant progression until end stage of disease was detected in *Nestin*-*Mfp2*^−/−^ mice (Supplementary Figure S1B). Furthermore, the delays in 34-weeks-old *Nestin*-*Mfp2*^−/−^ mice were still smaller in comparison to those in 17-weeks-old *Mfp2*^−/−^ mice. The substantial additional delay of the complete wave i.e., the 1–5 mean peak latency in *Mfp2*^−/−^ mice in comparison to *Nestin*-*Mfp2*^−/−^ mice was evident from 12 weeks of age (Figure 4O). The additional delay in *Mfp2*^−/−^ relative to *Nestin*-*Mfp2*^−/−^ mice was also confirmed by analysis of the interpeak latencies. Whereas 1–3 interpeak latencies were significantly increased in *Mfp2*^−/−^ mice vs. control mice, they were not altered in *Nestin*-*Mfp2*^−/−^ mice. No difference was observed for 3–5 interpeak latency in any of the genotypes (Figures 4J,M,P). Finally, peak amplitudes which indicate signal strength to an auditory stimulus were significantly reduced in *Mfp2*^−/−^ mice whereas *Nestin*-*Mfp2*^−/−^ mice showed intact wave amplitudes (Figures 4K,N,Q).

In conclusion, neuronal signal transmission is affected from the age of 6 weeks in brainstem of both *Mfp2^−/−^* and *Nestin*-*Mfp2*^−/−^ mice. However, brainstem responses are more severely affected in *Mfp2*^−/−^ mice and decline progressively coinciding with increased microgliosis.

### Differential Disturbances in Locomotion and Behavior in *Mfp2^−/−^* Compared to *Nestin-Mfp2^−/−^* Mice

**Figure 5:**
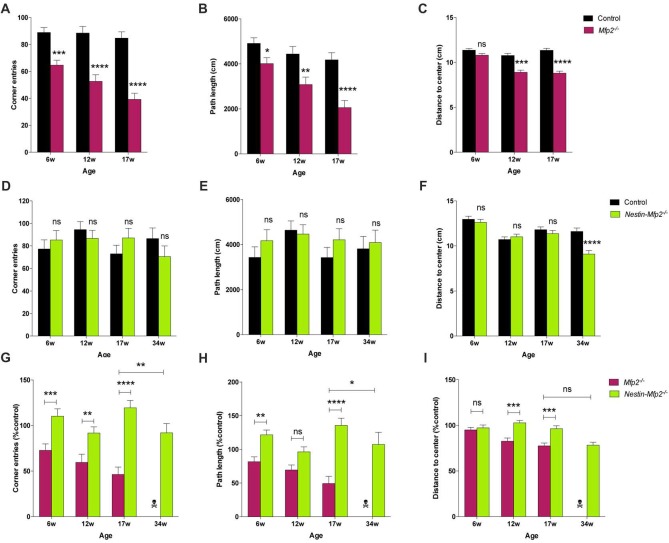
Early-onset disturbance in locomotor activity and exploration in *Mfp2^−/−^* mice vs. normal activity and late-onset disturbance of explorative behavior in *Nestin-Mfp2*^−/−^ mice. **(A,B,D,E)** Locomotor activity shown by corner entries **(A,D)** and total path length **(B,E)** was analyzed at onset, mid and end stage of disease in *Mfp2*^−/−^ mice **(A,B)** and *Nestin-Mfp2*^−/−^ mice **(D,E)** compared to control mice. **(C,F)** Explorative behavior at different disease stages in *Mfp2*^−/−^ mice **(C)** and *Nestin-Mfp2*^−/−^ mice **(F)**. **(G–I)** Direct comparison between *Mfp2*^−/−^ and *Nestin-Mfp2*^−/−^ mice relative to control mice. *Mfp2^−/−^ or *Nestin-Mfp2*^−/−^ vs. control **(A–F)**, *Nestin-Mfp2*^−/−^ vs. *Mfp2*^−/−^ **(G–I)**: *p < 0.05, **p < 0.01, ***p < 0.001, ****p < 0.001, ns, not significant. Error bars indicate SEM. *n* = 8–24 mice/group. 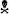
*Mfp2*^−/−^ mice all died at this time point.

The impact of global or neural-specific deletion of MFP2 in the brain on the clinical level such as motor abilities and behavior remains obscure. We analyzed locomotor activity and explorative behavior by open field (OF) test and extracted several parameters from video-tracks during exploration of mice in an OF environment. Total path length and corner crossings were included as measures of explorative locomotor activity. *Mfp2^−/−^* mice were less active at all ages, as indicated by reduced corner entries and total path length (Figures 5A,B). Locomotor activity in *Mfp2*^−/−^ mice declined between 6 and 17 weeks of age (Supplementary Figures S2A,B). In contrast, *Nestin*-*Mfp2*^−/−^ mice showed normal ambulation levels (Figures 5D,E) and no progression until end stage of pathology (Supplementary Figures S2D,E). Direct comparison of knockout models showed decreased locomotor activity in *Mfp2*^−/−^ in comparison to* Nestin*-*Mfp2*^−/−^ mice at all ages. Even 34-weeks-old *Nestin*-*Mfp2*^−/−^ mice were more active than 17-weeks-old *Mfp2*^−/−^ mice, indicated by both parameters (Figures 5G,H).

Mice prefer to walk along the walls of the arena with occasional traversal of the center. Exploration of the center was quantified by the average distance from the center as a measure of conflict resolution and anxiolysis, whereby control mice generally reside at the sides of the cage. Center exploration of the arena was normal in both models at 6 weeks of age (Figures 5C,F), but *Mfp2^−/−^* mice showed increased center exploration at 12 and 17 weeks of age despite their reduced locomotor activity (Figure 5C, Supplementary Figure S2C). This suggests progressive anxiolysis or a more general insensitivity to environmental cues. *Nestin*-*Mfp2*^−/−^ mice similarly developed a disproportionate exploration of the center, however only at the age of 34 weeks (Figure 5F, Supplementary Figure S2F).

These results indicate comparable emotional deficit in both knockout models upon approaching terminal stages of disease (Figure 5I). *Mfp2^−/−^* mice exhibit early-onset and progressive decline of motor functions whereas motor skills remain intact in *Nestin*-*Mfp2*^−/−^ mice.

### Grip Strength Is Decreased in *Mfp2^−/−^* and *Nestin-Mfp2^−/−^* Mice

**Figure 6:**
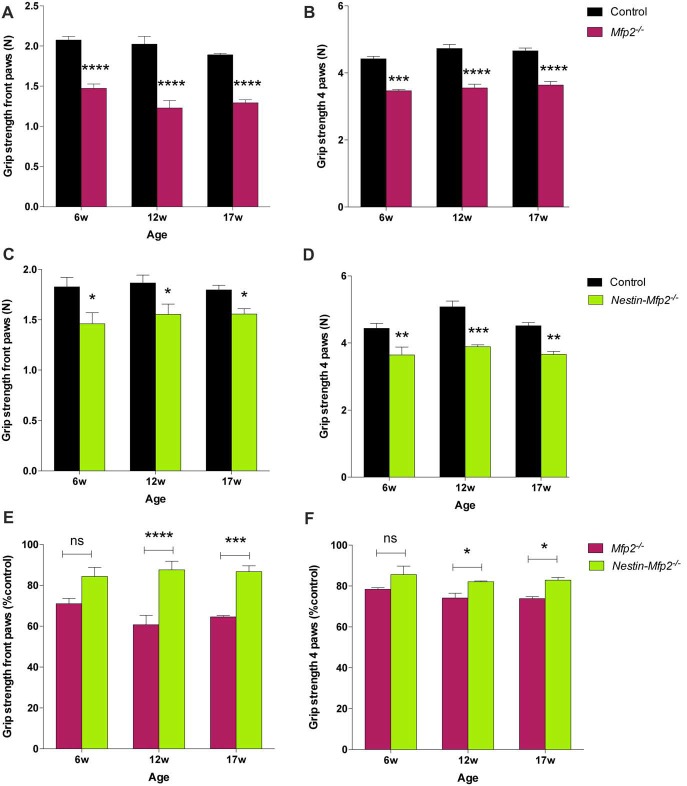
Grip strength is decreased in *Mfp2^−/−^* and *Nestin-Mfp2*^−/−^ mice. **(A–D)** Grip strength in front paws **(A,C)** and all paws **(B,D)** at different ages in *Mfp2*^−/−^ mice **(A,B)** and in *Nestin-Mfp2*^−/−^ mice **(C,D)**. **(E,F)** Direct comparison between the knockout models relative to control mice. Significance level: *p < 0.05, **p < 0.01 ***p < 0.001; ****p < 0.0001, ns, not significant. Error bars indicate SEM. *n* = 4–9 mice/group.

Both *Mfp2^−/−^* and *Nestin-Mfp2*^−/−^ mice develop motor problems from the age of 4 weeks that worsen with age and eventually lead to severe ataxia and tremor. We demonstrated previously that motor coordination by rotarod testing is impaired in both mouse models compared to control but is more severely affected in *Mfp2*^−/−^ compared to *Nestin-Mfp2*^−/−^ mice (Verheijden et al., 2013). Grip strength and coordination was assessed at different ages to reveal whether this was associated with the inflammatory state of the brain. Grip strength in both front and hind paws was decreased in 6-weeks-old *Mfp2*^−/−^ (Figures 6A,B) as well as in *Nestin-Mfp2*^−/−^ (Figures 6C,D) mice compared to age-matched controls. However, no clear progression until end stage of disease was detected. Comparison of both knockout mouse models showed that grip strength in front paws and all paws together was more severely affected in *Mfp2*^−/−^ mice compared to *Nestin-Mfp2*^−/−^ mice from 12 weeks of age, relative to their respective control mice (Figures 6E,F). Grip strength and motor coordination analysis by inverted grid test confirmed the results on both knockout models at all ages (Data not shown). We conclude that both *Mfp2*^−/−^ and *Nestin-Mfp2*^−/−^ mice show early-onset loss of grip strength which does not worsen with age.

## Discussion

The intent of the present study was to reveal which cellular dysfunctions underlie the striking difference in clinical decline between mice with constitutive and neural-selective deficiency of MFP2, the pivotal peroxisomal β-oxidation enzyme. We show here that development of microgliosis is delayed in *Nestin-Mfp2^−/−^* mice and that the genetically unaltered microglia achieve a minor activation profile regarding expression of inflammatory markers, loss of homeostatic markers and priming as compared to MFP2 deficient microglia in *Mfp2*^−/−^ mice. Normal locomotor activity and minor late-onset deficits in exploratory behavior and neuronal conductance in brain of *Nestin-Mfp2*^−/−^ mice accompanies the minor microgliosis. This is in sharp contrast with severe microgliosis and quick clinical deterioration of *Mfp2*^−/−^ mice, strongly suggesting that microgliosis accelerates deterioration of *Mfp2*^−/−^ mice.

Acute or chronic disruptions of CNS homeostasis are accompanied by morphological and functional microglial adaptations. It is becoming clear that no unifying concept can be defined whether this response is neuroprotective or neurodetrimental as this will largely depend on the molecular signature of microglia. We previously determined that microglia in *Mfp2^−/−^* mice acquire a highly proliferative activation state and chronically lose their homeostatic markers but that they are not overtly neurotoxic. Despite complete inactivation of MFP2 in all neural cells i.e., neurons, astrocytes and oligodendrocytes in *Nestin-Mfp2*^−/−^ mice (Verheijden et al., 2013), the microglial reaction was attenuated and delayed. As no peripheral pathologies and systemic inflammation nor neuronal loss and demyelination were detected in these mouse models, the amplified and worsening microglial reactivity is the only feature that parallels the early deterioration of *Mfp2*^−/−^ mice. Our data thus indicate that chronically inflammatory microglia worsen neurological dysfunction even if they are not polarized towards a clear pro-inflammatory state.

This was not only deduced from the more severe clinical deterioration in *Mfp2^−/−^* mice, but also by comparing neuronal functioning in both models. Neuronal markers that restrain microglia activation are downregulated in *Mfp2*^−/−^ mice from onset of disease whereas they remain unaffected in *Nestin-Mfp2*^−/−^ mice. In addition, we assessed BAEP evoked neuronal signal transmission in different brain regions such as brainstem and cortex where microgliosis initiates and is most pronounced during the disease course. Neuronal transmission of the auditory signal was affected in both mouse models but shows a clear additional delay in *Mfp2*^−/−^ compared to *Nestin*-*Mfp2*^−/−^ brain. The delay worsened during disease in *Mfp2*^−/−^ mice but not in *Nestin-Mfp2*^−/−^ mice. As there is no demyelination in cerebrum of *Mfp2*^−/−^ and *Nestin-Mfp2*^−/−^ mice (Verheijden et al., 2013), disturbed neuronal transmission cannot be attributed to loss of myelin (Shankar et al., 2000; Seeman et al., 2001). Besides transmission delay, signal amplitude is decreased in* Mfp2*^−/−^ but not in *Nestin-Mfp2*^−/−^ mice, which might be indicative of a defect at the first steps of auditory responding i.e., at the level of the cochlea or auditory nerve such as synaptopathy in the cochlea (Schaette and McAlpine, 2011; Mehraei et al., 2016). In view of the recent discoveries that microglia modulate synapse function and transmission in the brain, it is plausible that chronic loss of homeostatic microglial functioning contributes to neuropathological symptoms through effects on synapses (Schafer et al., 2013).

The progressive increase in microgliosis in *Mfp2^−/−^* mice, which is more pronounced than in *Nestin-Mfp2*^−/−^ mice at all ages, correlates with progressive abnormalities in locomotor activity and explorative behavior. *Mfp2*^−/−^ mice exhibit early-onset and progressive decline of locomotion whereas *Nestin-Mfp2*^−/−^ mice have a normal activity level and only develop altered explorative behavior prior to death. This suggests that *Mfp2*^−/−^ microglia in diseased brain play a detrimental role on neuronal functioning and behavior. We previously showed that *Mfp2*^−/−^ microglia do not adopt a typical neurotoxic state as several neurotoxic and pro-inflammatory markers including iNOS remain unaltered during disease. This sheds new light on novel signatures and corresponding functions of microglia in neuropathology.

In contrast to other behavioral deficits, grip strength is reduced from a young age in both knockout mouse models but does not worsen with time. These early-onset motor impairments in *Mfp2^−/−^* and *Nestin-Mfp2*^−/−^ mice are likely caused by neuronal-driven CNS pathologies as we previously showed that peripheral nerves, skeletal muscles and sensory conduction velocity in tail nerves remain intact in preterminal *Mfp2*^−/−^ mice (Huyghe et al., 2006c). We observed here that impaired grip strength does not exacerbate with expanding neuroinflammation. Ataxia and motor impairments in both mouse models might stem from cerebellar pathology due to Purkinje cell dysfunction, which we recently demonstrated in *Nestin-Mfp2*^−/−^ mice (Verheijden et al., 2013).

We hypothesize that the pathological events in the MFP2 deficient mouse brain are initiated by dysfunctional neurons. This is supported by the fact that neuroinflammation is confined to gray matter regions. On top, inherent loss of MPF2 in microglia might cause an exaggerated response to primary neural deficits, via cell-autonomous mechanisms. Neural-microglial bidirectional communication dysfunction may synergistically lead to rapid deterioration in *Mfp2^−/−^* mice. This is supported by downregulation of neuronal signals that restrain microglial activation in *Mfp2*^−/−^ but not in *Nestin*-*Mfp2*^−/−^ brain, which coincides with aggravation of microgliosis in *Mfp2*^−/−^ brain. To support this hypothesis, further analysis of cell-autonomous contribution of *Mfp2*^−/−^ microglia to disease is necessary e.g., by development of a mouse model with microglia restricted loss of MFP2.

To date, it remains unexplored whether similar mechanisms are at play in the gray matter of MFP2 patients. It would be instructive to visualize neuroinflammation in MFP2 deficient patients by performing positron emission tomography using a TSPO ligand. In *Mfp2^−/−^* mice we previously showed that this imaging marker for neuroinflammation is upregulated during disease (Beckers et al., 2018).

In conclusion, we propose that the chronic highly inflammatory state of *Mfp2^−/−^* microglia worsens the initial neuropathology caused by absence of MFP2 from neural cells and accelerates clinical deterioration in MFP2 deficiency.

## Author Contributions

LB and MB: conceived study. LB: experimental design, performed all experiments, analyzed data, made figures and wrote the manuscript. SS: statistical analysis and interpretation of BAEP and OF tests. RD: scientific input and interpretation of results related to BAEP and OF tests. MB: design, editing of figures and manuscript.

## Conflict of Interest Statement

The authors declare that the research was conducted in the absence of any commercial or financial relationships that could be construed as a potential conflict of interest.

## Supplementary Material

FIGURE S1 Neuronal dysfunction progressively worsens in Multifunctional protein-2 (*Mfp2^−/−^*) but not in *Nestin-Mfp2*^−/−^ mice. **(A,B)** Mean peak latencies at onset and end stage of disease in *Mfp2*^−/−^ mice **(A)** and in *Nestin-Mfp2*^−/−^ mice **(B)** relative to age-matched control mice. **p* < 0.05, *****p* < 0.0001, ns, not significant. Error bars indicate SEM. *n* = 11–15 mice/group.

FIGURE S2 Progressive decline of motor skills and emotional behavior in *Mfp2*^−/−^ mice vs. intact locomotion and preterminal onset of emotional deficit in *Nestin-Mfp2*^−/−^ mice. **(A–F)** Progression of locomotor activity **(A,B,D,E)** and emotional deficit **(C,F)** between onset (6 weeks) and preterminal stage of disease in *Mfp2*^−/−^ mice **(A–C)** and *Nestin-Mfp2*^−/−^ mice **(D–F)**. *6 weeks (6 w) vs. preterminal stage: **p* < 0.05, ****p* < 0.001, ns, not significant. Error bars indicate SEM. *n* = 8–24 mice/group.
