# Supplementary figures and images for: Neuronal Dysfunction and Behavioral Abnormalities Are Evoked by Neural Cells and Aggravated by Inflammatory Microglia in Peroxisomal β-Oxidation Deficiency
Source: Front Cell Neurosci. 2018 May 23;12:136. doi: 10.3389/fncel.2018.00136 (PMC5975114; doi:10.3389/fncel.2018.00136)

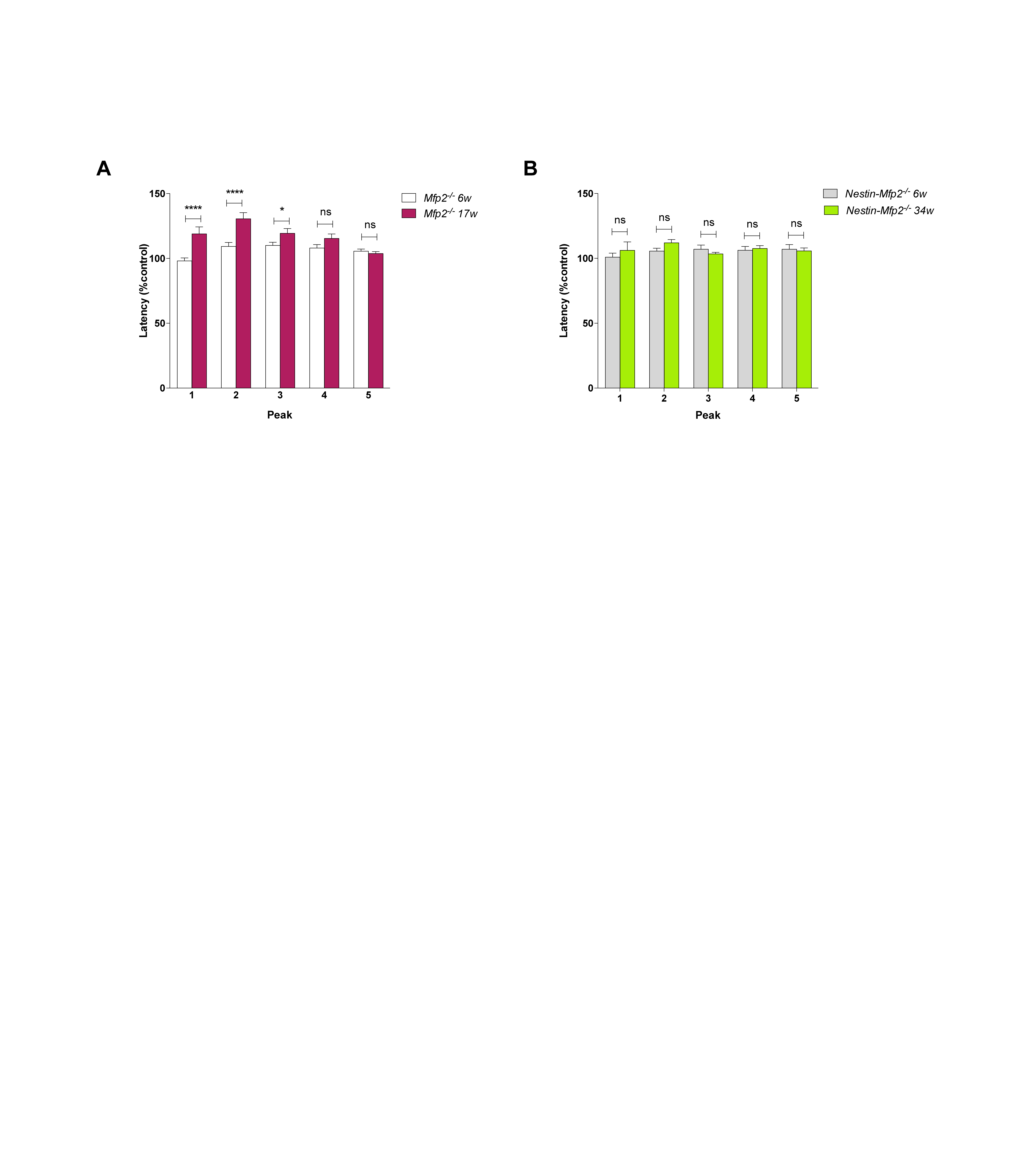

Supplement: FIGURE S1 — Neuronal dysfunction progressively worsens in Multifunctional protein-2 (Mfp2−/−) but not in Nestin-Mfp2−/− mice. (A,B) Mean peak latencies at onset and end stage of disease in Mfp2−/− mice (A) and in Nestin-Mfp2−/− mice (B) relative to age-matched control mice. *p < 0.05, ****p < 0.0001, ns, not significant. Error bars indicate SEM. n = 11–15 mice/group. [file Image_1.TIF]
